# Supplementary material for: Angelica sinensis polysaccharide as potential protectants against recurrent spontaneous abortion: focus on autophagy regulation
Source: Front Med (Lausanne). 2025 Jan 15;12:1522503. doi: 10.3389/fmed.2025.1522503 (PMC11774876; doi:10.3389/fmed.2025.1522503)
Supplement: Supplementary material S1 — The main instruments used during the LC-MS process, along with their models/specifications and manufacturers. [file Supplementary_file_1.zip › Metabolomics sequencing data FC1.2/8.代谢通路分析/Exposure_Ctrl/all/KEGG/mmu00052.html]

KEGG PATHWAY: Galactose metabolism - Mus musculus (house mouse)


# Galactose metabolism - Mus musculus (house mouse)


[
Pathway menu
| Organism menu
| Pathway entry
| Download
| Help
]


##### Option

Scale:


100%

Image resolution:


 High

##### Search

##### ID search

##### Color

##### Module

Complete only
Including 1 block missing
Including any incomplete

- Pathway modules
  - Carbohydrate metabolism
    - Central carbohydrate metabolism
      - M00633
        Semi-phosphorylative Entner-Doudoroff pathway
      - M00309
        Non-phosphorylative Entner-Doudoroff pathway
    - Other carbohydrate metabolism
      - M00632
        Galactose degradation, Leloir pathway
      - M00552
        D-galactonate degradation, De Ley-Doudoroff pathway
      - M00554
        Nucleotide sugar biosynthesis


KGML

Image (png) file 1x
